# Random thoughts on pediatric surgery in India

**DOI:** 10.4103/0971-9261.43013

**Published:** 2008

**Authors:** Sujit K. Chowdhary

**Affiliations:** Senior Consultant (Ped Urology & Ped Surgery) Indraprastha Apollo Hospital, New Delhi - 110076, India. E-mail: sujitchowdhary@hotmail.com

Pediatric surgery is the youngest subspecialty of surgery. In India, it has continued to develop since its initiation four decades ago by the pioneers like U. C. Chakraborty, Raman Nair, O. Anjaneyulu, R. K. Gandhi, I. C. Pathak, Subir Chatterjee, M. S. Ramakrishnan, K. C. Sogani, P. Upadhyaya, T. Dorairajan and S. S. Deshmukh. However, in the present world, the market value of a specialty in surgery is directly proportional to the job opportunities available for those who have completed training and annual earning of a surgeon in that speciality. This is indirectly reflected by the quality and number of surgical postgraduates seeking entry into the specialty. In recent times, reportedly, many of recognized training units have failed to recruit a candidate !. However the Medical Council of India and the National Board of Examinations have become very liberal in granting accreditations. Similar to most super specialties, there is an acute concentration of pediatric surgeons in big cities; many feel that still there is plenty of clinical material for the pediatric surgeons at the district level in our country. Ours is a child-centric society, and yet the lack of awareness and poverty play a crucial role in preventing the vast majority of rural children from accessing quality pediatric surgical care.

In sharp contrast, until recently, in United States, pediatric surgeons had comparable job opportunities and earnings as cardiovascular surgeons. In a developing nation, there are immense opportunities as well as problems! If IAPS can plan and execute the plan for growth of pediatric surgery, it will become a specialty with immense opportunities here too! On the basis of the extreme hardship and sincere efforts invested by the grandmasters of the specialty for over long years, the specialty is beginning to gain public recognition.

Pediatric surgery is an extensive specialty in which one aims to become an expert in the surgery of every organ of the body. Whereas, it is a great aim, in practice with the fast-moving cutting-edge technology, changing health care structures, expanding medico legal implications in private practice, etc. Such an aim would sooner or later appear self-limiting, unless one is in a state-run-protected market. The majority of new pediatric surgeons have to face much tougher market conditions than their predecessors, albeit with new opportunities.

Neonatal surgery is our flagship subspecialty. The majority of MCh training centers are struggling with 35% mortality for complex neonatal surgeries. That picture has to change without delay. If a premature or small-for-date baby dies after a surgery because of the lack of supportive care, then this does not serve as a sufficiently satisfactory explanation. Every unit has to introspect, audit and obtain the survival figures up to greater than 90%, which is the acceptable standard in any specialty of surgery that means business.

The leaders of IAPS and program directors will have to deliberate on the future they desire for pediatric Surgeons and the specialty of pediatric surgery. In the same public institute, a surgeon possessing the skill to safely excise a choledochal cyst and perform a major gastrointestinal reconstruction is perceived as a generalist, whereas the one diagnosing a choledochal cyst is usually treated as a specialist by the public, general physicians, pediatricians and even surgeons!

Skill acquisition, absorption of technology and progress of knowledge are all related to a number game; this is provided the turf on which professional practices have the appropriate facility. That is where the role of professional regulation enters the game. Ours is a specialty with a prevalence of common conditions, i.e., inguinal hernia in the 1:100 live births and hard core specialty conditions such as choledochal cyst in 1:3000 live births. The projected western figures often quoted in the Indian literature to justify the rapid production of pediatric surgeons in India are meaningless for several reasons, i.e., an unregulated market, an immature regulatory system, the lack of insurance cover for congenital malformations, etc.

The common general surgical problems in babies and children have been effectively dealt by general surgeons and will remain that way. Our country is very vast, and pediatric surgeons with current training arrangement will continue to be an asset to the rural population and peripheral towns, thereby bringing new standards of surgical care for babies and children hitherto nonexistent.

The calculation for the requirement for 2000 pediatric surgeons for the country on the basis of the population data of the country and western logistic planning is flawed. Our economy is growing and will still need 30–40 years of sustained growth for health planning to be extrapolated as per western studies. In our context, in general, 20 established pediatricians are required to provide professional work to one pediatric surgeon to keep him/her active. If this fact is true, the right numbers have already been reached! That is probably the reason for slow down in the market value and sluggish entry of surgical postgraduates into this specialty.

Pediatric surgeons wishing to practice in the expanding corporate structures and first-line university hospitals will have to train harder and longer in the specialized areas within pediatric surgery, i.e., urology, gastrointestinal surgery, thoracic surgery, neurosurgery, transplantation, etc. They are to then take up specialized practice within a group to prove their merit and earn recognition for what they capable of performing. Each of such surgeons will have to continue performing general pediatric surgery and neonatal surgery until the work volume expands to keep the person doing just that specialty work. By 2020, there are likely to be 20 additional positions in the corporate structure alone in the country, where such subspecialty-trained surgeons will have job opportunities.

Where will these surgeons train? That is where the role of key training departments lies. The country has made large investments in the departments of AIIMS, PGI, CMC, etc., and it is not possible to duplicate such departments elsewhere overnight! These departments will have to start advanced pediatric surgery subspecialty programs in specialized areas that will imply a few more years of grind for the trainee. The subspecialty training program could be even more meaningful and relevant if public and private institutions can participate in it. The additional training should be followed by accreditation by a subspecialty board constituted by IAPS.

A great deal has been achieved; as always, the climb gets tougher as one goes higher! The future of pediatric surgery and surgeons will be much stronger and brighter if IAPS is empowered and it truly starts to operate as a professional national organization beyond partisan interests. If these developments are executed, the best days for pediatric surgery are yet to arrive!

